# Convergent validity of ActiGraph and Actical accelerometers for estimating physical activity in adults

**DOI:** 10.1371/journal.pone.0198587

**Published:** 2018-06-12

**Authors:** Scott Duncan, Tom Stewart, Mikkel Bo Schneller, Suneeta Godbole, Kelli Cain, Jacqueline Kerr

**Affiliations:** 1 Human Potential Centre, Auckland University of Technology, Auckland, New Zealand; 2 Research Unit for Active Living, University of Southern Denmark, Odense, Denmark; 3 Steno Health Promotion Research, Steno Diabetes Center, Gentofte, Denmark; 4 Department of Family Medicine and Public Health, University of California, San Diego, United States of America; University of Tennessee Health Science Center, UNITED STATES

## Abstract

**Purpose:**

The aim of the present study was to examine the convergent validity of two commonly-used accelerometers for estimating time spent in various physical activity intensities in adults.

**Methods:**

The sample comprised 37 adults (26 males) with a mean (SD) age of 37.6 (12.2) years from San Diego, USA. Participants wore ActiGraph GT3X+ and Actical accelerometers for three consecutive days. Percent agreement was used to compare time spent within four physical activity intensity categories under three counts per minute (CPM) threshold protocols: (1) using thresholds developed specifically for each accelerometer, (2) applying ActiGraph thresholds to regression-rectified Actical CPM data, and (3) developing new ‘optimal’ Actical thresholds.

**Results:**

Using Protocol 1, the Actical estimated significantly less time spent in light (-16.3%), moderate (-2.8%), and vigorous (-0.4%) activity than the ActiGraph, but greater time spent sedentary (+20.5%). Differences were slightly more pronounced when the low frequency extension filter on the ActiGraph was enabled. The two adjustment methods (Protocols 2 and 3) improved agreement in this sample.

**Conclusions:**

Our findings show that ActiGraph and Actical accelerometers provide significantly different estimates of time spent in various physical activity intensities. Regression and threshold adjustment were able to reduce these differences, although some level of non-agreement persisted. Researchers should be aware of the inherent limitations of count-based physical activity assessment when reporting and interpreting study findings.

## Introduction

Physical inactivity has become a prominent area of research because of its known associations with chronic disease [1]. Indeed, increasing the proportion of the population who meet physical activity guidelines is a public health priority in many countries [2]. Accurate assessment of physical activity is therefore crucial for identifying important dose-response relationships with health outcomes, and understanding current physical activity patterns and practices. This information is necessary for the development of physical activity guidelines, interventions, and policy recommendations to promote physical activity and improve population health [3].

Measuring physical activity objectively has gained rapid traction as it eliminates many of the problems associated with self-reported information, and provides a stronger evidence base for health promoters [4, 5]. Wearable motion sensors, known as accelerometers, have been popularised as a measurement instrument over the last decade, as they are able to provide an accurate and reliable assessment of activity behaviour in free-living conditions. These devices are typically small-sized and discreet, making them well-suited for monitoring over an extended period. A number of purpose-built accelerometers are now commercially available, yet data comparability between them is limited, which is attributable to differences in their electro-mechanical design, and how the raw acceleration signals are processed [6, 7].

Commercially available accelerometers come equipped with different types of sensors (e.g., piezoelectric or capacitive sensors) which measure acceleration slightly differently, but all are based on the principle of generating a signal that is directly proportional to the acceleration detected [6]. These electrical signals are normally amplified, digitised, rectified and filtered, before being converted into an arbitrary unit known as an activity ‘count’. These counts are a representation of movement intensity over a specified time period (i.e., an epoch), with a higher count indicating higher intensity. A set of thresholds–commonly referred to as ‘cut points’–are then applied to the data, whereby each record in the dataset is assigned to one of four physical activity intensity categories; sedentary, light, moderate, or vigorous [8]. These data are then aggregated and time spent in each intensity category is obtained. This information can then be used to check adherence to physical activity guidelines (i.e., time spent in moderate or vigorous intensity physical activity each day).

However, data obtained post-processing (i.e., activity counts) may not truly represent the raw acceleration signals from which they were derived. This is because of the signal transformation techniques used by each manufacturer and how the activity counts are obtained. Converting a filtered signal into an activity count is achieved via a closely guarded proprietary algorithm that varies by manufacturer. This means that data obtained from different accelerometer brands and models are generally not comparable; equivalent activity counts across two different devices may not necessarily represent the same activity intensity [9]. Consequently, comparing count-based results across different studies, or pooling data from different sources is not possible without applying a correction equation to align the two data streams [7]. The obscurity surrounding how counts are obtained, and what they represent in reality, has meant activity recognition from raw acceleration signals is starting to take precedence over count-based measures. Universal processing methods are being developed which can be applied to raw acceleration signals from a range of accelerometer brands [10]. While this may be a step in the right direction, many older model accelerometers that have been used extensively over the last decade (representing the majority of evidence in this field) do not possess the ability to output raw unfiltered acceleration information and must make use of count-based estimates of physical activity and energy expenditure.

To date, the two most popular accelerometer brands used in this field are the ActiGraph (ActiGraph Inc, Pensacola, FL) and Actical (Phillips Respironics, Bend, OR). A series of controlled lab tests revealed the activity counts per minute (CPM) output from these two devices were incomparable [9]. Even in free-living conditions, the ActiGraph–both the original 7164 and the newer GT3X model–are known to record up to 20% more CPM compared to the Actical after multiday assessment periods [11, 12]. In addition, Evenson et al [13] reported that the Actical is relatively insensitive when monitoring low intensity activity (< 200 cpm), and uses a key set of values recorded repetitively rather than recording the ‘true’ value on a continuous scale. Nonetheless, the key determinant of agreement between devices is their ability to provide equivalent information about physical activity or energy expenditure [7]. Although CPM is a measure of average activity over a one-minute period, it does not represent the time spent within different intensity categories over an extended period (a metric used to assess adherence to physical activity guidelines). Having focused exclusively on direct CPM comparisons, previous device-comparison studies have overlooked the possibility that device-specific CPM thresholds may rectify CPM incongruence by correctly classifying time spent within each activity intensity category.

In this study we propose to test the convergent validity of the ActiGraph GT3X+ and the original Actical accelerometer, by assessing the agreement between units when classifying time spent within four physical activity intensity categories under a number of CPM threshold conditions. This will be achieved by (1) using thresholds developed and validated specifically for each accelerometer, (2) applying ActiGraph thresholds to regression-rectified Actical CPM data, and (3) developing new ‘optimal’ Actical thresholds based on alignment with the ActiGraph data.

## Materials and methods

### Participants

A convenience sample of 37 adults (26 males) with a mean (SD) age of 37.6 (12.2) years (range: 23–56) was recruited from the University of California, San Diego, USA. Participants were recruited in November 2011 from a university campus and self-identified as commuter cyclists. The characteristics of the sample are presented in Table 1. All participants from provided written informed consent prior to being involved in the study, and ethical approval was obtained from the Institutional Review Board at UCSD before the study commenced. All data are from this study are included as Supporting Information (S1 Table).

### Instruments

Actical Model B (AC; Philips Healthcare, Andover, MA) and ActiGraph GT3X+ (AG, Pensacola, FL) accelerometers were used in this study. The AC is a motion sensor equipped with an omnidirectional piezoelectric accelerometer. While the sensor is omnidirectional, it is most sensitive to movement in the vertical plane when worn on the hip, due to the placement of the sensor within the device [6]. The acceleration signals are digitised at a set sampling frequency of 32 Hz. However, obtaining raw data from the AC is not possible–the minimum user-defined epoch length is 15 seconds–which must be set before data collection commences [6].

The AG GT3X+ is a newer triaxial capacitive accelerometer capable of collecting and recording movement at a user-defined sampling frequency of up to 100 Hz [6]. Unlike the AC, the AG has the ability to store raw data collected at the selected sampling frequency. Raw AG acceleration signals can be processed using one of two filters; the normal filter, or the low frequency extension (LFE) filter. According to ActiGraph, the LFE filter permits lower intensity movements to be detected, so may be useful for slow moving populations such as the elderly. It has been shown that the LFE filter maintains comparability with earlier ActiGraph models in adults, but the low sensitivity threshold renders step counts unusable [14]. However, the extent to which the selected filter influences the agreement with other devices is currently unknown. The AG’s ability to store raw information means data can be reintegrated into different epoch lengths using different filters post collection, enabling the comparison of different filters.

### Procedures

Height and weight were measured using standardised protocols. Body Mass Index (BMI) was calculated as weight (kg) divided by height (m) squared. The AC was setup to record 60 s epochs at a sampling frequency of 32 Hz, and the AG was set to log raw data at 30 Hz. Both devices were initialised on the same computer to synchronise their internal clocks. Each participant was fitted with two devices on an elastic waist belt; the AG was placed over the right anterior superior iliac spine and the AC was placed alongside it. Participants wore both devices over a 3-day monitoring period, and were encouraged to go about their normal daily routines. Each participant was instructed to wear the devices at all times, only removing them before sleeping, or when they may come in contact with water (e.g., swimming). Upon completion of the 3-day monitoring period, all devices were collected by the respective research teams.

### Data treatment

The AC 60 s epoch data were downloaded using Actical Software (version 2.1; Mini Matter, Bend, OR). Raw AG data were downloaded using Actilife (version 6; ActiGraph, Pensacola, FL) before being reintegrated into 60 s epochs using the normal filter, and again with the LFE filter enabled. This resulted in three data streams across both devices: AC, AG_NORM_, and AG_LFE_. The vector magnitude–a composite of counts from each of the three axes–were used for the AG CPM data for comparability with the omnidirectional sensor present in the AC. All data were pooled, and non-wear time–classified as 30 minutes of continuous zero counts in either data stream–was removed before scoring the data with intensity thresholds. Three protocols were developed to assess how different CPM threshold conditions classified time spent at different physical activity intensities, and how these estimates varied between devices. The following three protocols were designed by treating the AG as the criterion; the AC CPM data or threshold values were manipulated while the AG CPM data and thresholds were held constant.

#### Protocol 1 –Using validated device-specific CPM thresholds

Both the AC and the AG have established CPM intensity thresholds for sedentary, light, moderate, and vigorous intensity physical activity. These intensity classifications correspond to commonly employed metabolic equivalent of task (MET) categories and are typically developed in the laboratory by walking and running at different speeds on a treadmill. In adults, frequently used thresholds for the AG and AC are those developed by Freedson et al [15] (sedentary: ≤ 99 cpm; light: 100–1951 cpm; moderate: 1952–5724 cpm; vigorous: ≥ 5725 cpm) and Colley & Tremblay [16] (sedentary: ≤ 99 cpm; light: 100–1534 cpm; moderate: 1535–3961 cpm; vigorous: ≥ 3962 cpm), respectively. These AG thresholds were applied to both the AG_NORM_ and AG_LFE_ data streams, while the AC thresholds were applied to AC data. From this, the minutes spent in each of the four intensity categories were obtained.

#### Protocol 2 –Converting AC CPM to AG CPM via regression

Using regression to realign CPM information from different devices is an approach which has been used previously [11, 12]. In the present study, two linear mixed effect models were used to convert the AC CPM to predicted AG_NORM_ CPM and AG_LFE_ CPM values, respectively, before scoring the data with the established AG thresholds described in Protocol 1. The use of mixed effect models enabled individual subject differences to be included as random effects (intercept only). Neither timestamp nor day of study were significant random effects and were not included in the final models. In both models, residuals deviated significantly from normality, and therefore a square root transformation was applied. Models were developed in non-zero data only to ensure the overall intercept was not bound to zero, resulting in a better fit to the data. In other words, all AC data scored as zero remained as such in the predicted values, with the following equations applied to any CPM value of 1 or greater:

sqrt(AGNORM)=0.95xsqrt(AC)+15.52

sqrt(AGLFE)=0.94xsqrt(AC)+17.87

#### Protocol 3 –Developing ‘optimal’ Actical thresholds based on ActiGraph data

An alternative to converting raw CPM values between devices is to adjust the threshold values that define each intensity. To investigate the comparative effectiveness of this approach, a custom script was written in SAS (v9.4, SAS Institute, North Carolina, NC) to calculate the within-subject bias and correlation in the percentage of time spent within each intensity category for a range of potential AC thresholds (using the aforementioned AG thresholds as the criterion). For practical reasons, the potential thresholds were tested in 10 count increments. A linear regression was applied to the data after threshold adjustment, and the bias in the predicted values were assessed. The thresholds that resulted in the best balance between low mean bias and high correlation for the percentage of time spent in each intensity category were selected as the optimal thresholds. In instances where the lowest mean bias and the highest correlation did not occur at the same threshold value, obtaining the lowest possible mean bias was given priority. The use of these two statistical indices ensured that the adjusted AC thresholds were based on practical considerations of how data will be used in the field (i.e., to investigate the physical activity levels of individuals). This process was repeated twice, once using the AG_NORM_ data as the criterion, and then using the AG_LFE_ data as the criterion. The AC data were scored with the following calculated intensity thresholds to establish time spent in each intensity category:

AGNORM:Sedentary<1;Light1−729;Moderate730−3399;Vigorous≥3400.

AGLFE:Sedentary<1;Light1−579;Moderate580−3249;Vigorous≥3250.

### Statistical analyses

Descriptive statistics were generated using SAS (v9.4, SAS Institute, North Carolina, NC). Differences in participant characteristics (age, height, weight, and BMI) were assessed between sexes using independent-samples t-tests. Differences in the CPM values among the three accelerometer data streams (AC, AG_NORM_, AG_LFE_) were assessed using repeated measures ANOVA. Differences in the percentage of time spent in each intensity category were evaluated using Bland-Altman techniques. The effects of threshold adjustment or CPM conversion on the agreement among the intensity estimates derived from AG and AC were assessed using percent agreement (minutes classified ‘correctly’ divided by the total number of minutes classified). Cohen’s kappa was used to assess the strength of agreement between devices, which adjusts for the proportion of agreements which occur by chance, and has been recommended for equivalency studies of this nature [7]. Magnitude thresholds for kappa were based on Landis & Koch [17]. A related-samples McNemar test (binomial distribution) was used to test for differences in the proportions of correctly classified activity within each intensity category.

## Results

The descriptive characteristics of the sample are presented in Table 1. Differences in height and weight were observed between males and females. The mean wear time per day was 11.2 ± 2.1 hours. The average CPM was higher for the AG_NORM_ (596 ± 203) and AG_LFE_ (662 ± 219) than AC (236 ± 94). Between-device variation was consistent in both males and females.

**Table 1:** Characteristics of the sample.

	Male	Female	All
n	26	11	37
Age, yr	36.8 (12.4)^1^	39.7 (11.8)	37.6 (12.2)
Height, m	1.80 (0.07)	1.66 (0.05)	1.76 (0.09)
Weight, kg	79.8 (13.8)	59.0 (4.7)	73.6 (15.2)
Body mass index, kg.m^-2^	24.5 (3.3)	21.3 (1.5)	23.6 (3.2)
ActiGraph_Norm_, CPM	611 (223)	560 (153)	596 (203)
ActiGraph_LFE_, CPM	681 (241)	619 (163)	662 (219)
Actical, CPM	246 (103)	214 (71)	236 (94)

^1^Data are presented as means (SD).

**Fig 1:**
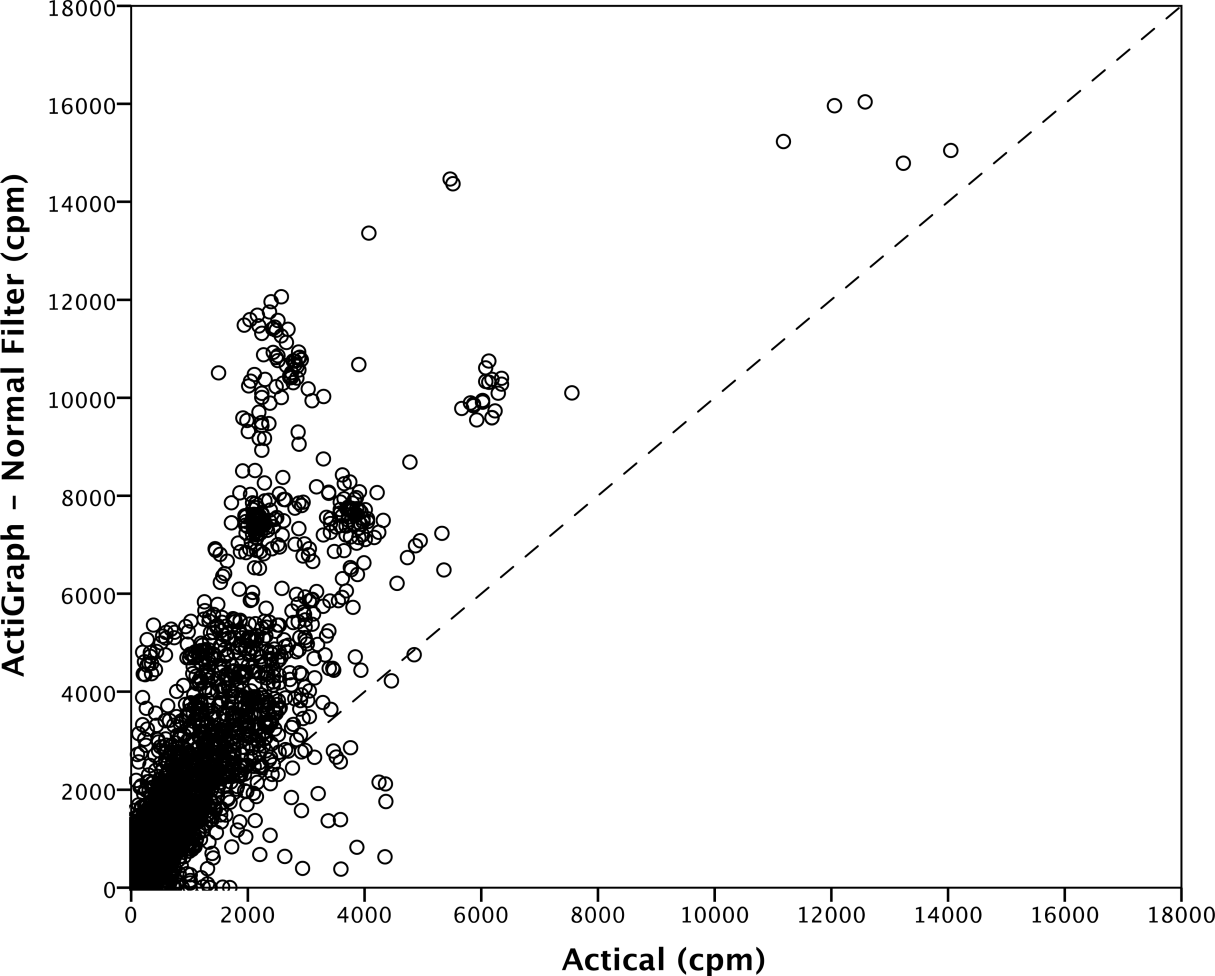
Scatterplot depicting the relationship between ActiGraph (normal filter) and Actical counts per minute (CPM) (r = 0.815).

Fig 1 provides an illustration of the differences between AG_NORM_ and AC CPM estimates. In general, AG_NORM_ tended to record higher CPM across the intensity spectrum compared to the AC. However, there were clusters of data at higher CPM values wherein AC tended to overestimate AG_NORM_. There were also five extreme values with CPM greater than 10,000; while the correlation coefficient increased from 0.815 to 0.845 when these values were removed, the values were retained in subsequent analysis as they were similar between devices and therefore unlikely to be the result of instrument error. The AG_LFE_ data showed identical visual trends and were not presented here.

**Fig 2:**
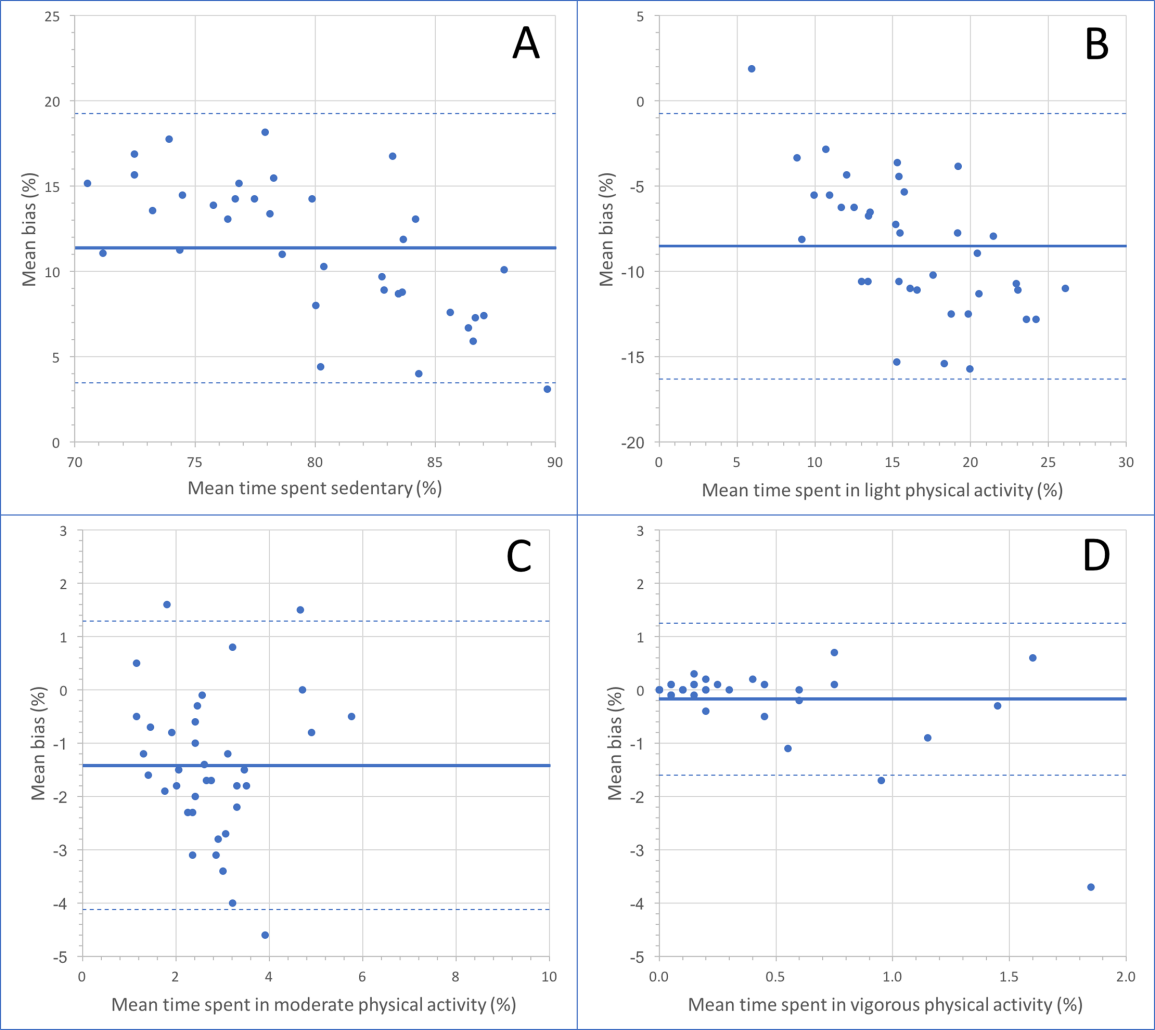
Bland-Altman plots comparing ActiGraph (normal filter) and Actical estimates of time spent in (A) sedentary, (B) light, (C) moderate, and (D) vigorous physical activity.

Fig 2 shows the Bland-Altman plots used to demonstrate differences in the percentage of time spent in each physical activity intensity recorded by the AC and AG_NORM_. Mean percent bias (95% limits of agreement) were 11.4 (3.48, 19.3), -8.51 (-16.3, -0.729), -1.42 (-4.12, 1.28), and -0.176 (-1.60, 1.25) for sedentary, light, moderate, and vigorous physical activity, respectively. In other words, when compared to AG_NORM_, AC overestimated time spent in sedentary behaviour, underestimated time spent in light physical activity, with minimal bias for moderate and vigorous physical activity.

The minute-by-minute agreement between the AC and AG_NORM_, and the AC and AG_LFE_, for each of the three protocols, are presented in Table 2 and Table 3, respectively. When the device-specific thresholds were used in Protocol 1, the AC recorded at least 20% more sedentary time, and at least 16% less light intensity PA than the AG_NORM_ and the AG_LFE_. This trend was also present for the second and third protocols, although the differences were not as pronounced. At higher intensities, the difference between devices was less substantial, with absolute frequency differences ranging from 0.4% to 6.8%.

**Table 2:** Minute-by-minute agreement between ActiGraph (normal filter; Freedson et al cut points [15]) and Actical using three different protocols. The highest kappa statistic(s) for each intensity level is bolded.

	Percentage (diff from AG_NORM_)	Agreement	κ	Magnitude	P_diff_^1^
*Protocol 1*: Default AC thresholds					
Sedentary (< = 99 cpm)	73.3% (+20.5%)	77.6%	0.541 ± 0.002	Moderate	< 0.001
Light (100–1534 cpm)	21.8% (-16.3%)	72.9%	0.374 ± 0.003	Fair	< 0.001
Moderate (1535–3961 cpm)	3.6% (-2.8%)	93.1%	0.275 ± 0.006	Fair	< 0.001
Vigorous (> = 3962 cpm)	0.5% (-0.4%)	99.2%	**0.471 ± 0.016**	Moderate	< 0.001
MVPA (> = 1535 cpm)	4.1% (-5.1%)	94.3%	0.547 ± 0.005	Moderate	< 0.001
*Protocol 2*: Converted CPM values^2^					
Sedentary (< = 99 cpm)	56.6% (+3.8%)	88.9%	**0.777 ± 0.002**	Substantial	< 0.001
Light (100–1951 cpm)	36.1% (-2.0%)	84.1%	0.660 ± 0.002	Substantial	< 0.001
Moderate (1952–5724 cpm)	6.8% (+0.4%)	93.2%	**0.446 ± 0.005**	Moderate	< 0.001
Vigorous (> = 5725 cpm)	0.5% (-0.4%)	99.2%	0.468 ± 0.016	Moderate	< 0.001
MVPA (> = 1952 cpm)	7.3% (-1.9%)	95.0%	**0.666 ± 0.004**	Substantial	< 0.001
*Protocol 3*: Optimal AC thresholds^3^					
Sedentary (< 1 cpm)	55.8% (+3.0%)	88.4%	0.767 ± 0.002	Substantial	< 0.001
Light (1–729 cpm)	36.5% (-1.6%)	84.1%	**0.661 ± 0.002**	Substantial	< 0.001
Moderate (730–3399 cpm)	6.5% (+0.1%)	93.1%	**0.446 ± 0.005**	Moderate	< 0.001
Vigorous (> = 3400 cpm)	0.3% (-0.6%)	99.3%	0.439 ± 0.017	Moderate	< 0.001
MVPA (> = 730 cpm)	6.9% (-2.3%)	95.0%	0.662 ± 0.004	Substantial	< 0.001

^1^Related-samples McNemar test (binomial distribution).

^2^CPM conversion equation [sqrt(CPM_AG_) = 0.95 x sqrt(CPM_AC_) + 15.52] applied in conjunction with default ActiGraph cut-points. Equation applied to non-zero data only.

^3^Optimal cut-points for predicting ActiGraph intensities with normal filter activated.

**Table 3:** Minute-by-minute agreement between ActiGraph (LFE filter; Freedson et al cut points [15]) and Actical using three different protocols. The highest kappa statistic(s) for each intensity level is bolded.

	Percentage (diff from AG_LFE_)	Agreement	κ	Magnitude	P_diff_^1^
*Protocol 1*: Default AC thresholds					
Sedentary (< = 99 cpm)	73.3% (+24.2%)	74.1%	0.486 ± 0.002	Moderate	< 0.001
Light (100–1534 cpm)	21.8% (-18.2%)	68.0%	0.279 ± 0.003	Fair	< 0.001
Moderate (1535–3961 cpm)	3.6% (-6.3%)	92.3%	0.399 ± 0.005	Fair	< 0.001
Vigorous (> = 3962 cpm)	0.5% (-0.6%)	99.2%	0.470 ± 0.015	Moderate	< 0.001
MVPA (> = 1535 cpm)	4.1% (-6.8%)	92.8%	0.489 ± 0.005	Moderate	< 0.001
*Protocol 2*: Converted CPM values^2^					
Sedentary (< = 99 cpm)	56.6% (+7.5%)	87.4%	**0.748 ± 0.002**	Substantial	< 0.001
Light (100–1951 cpm)	35.0% (-5.0%)	81.6%	**0.609 ± 0.003**	Moderate	< 0.001
Moderate (1952–5724 cpm)	7.8% (-2.1%)	93.3%	0.584 ± 0.004	Moderate	< 0.001
Vigorous (> = 5725 cpm)	0.6% (-0.5%)	99.1%	**0.482 ± 0.015**	Moderate	< 0.001
MVPA (> = 1952 cpm)	8.4% (-2.5%)	94.0%	0.659 ± 0.004	Substantial	< 0.001
*Protocol 3*: Optimal AC thresholds^3^					
Sedentary (< 1 cpm)	55.8% (+6.7%)	86.9%	0.739 ± 0.002	Substantial	< 0.001
Light (1–579 cpm)	34.5% (-5.5%)	81.6%	**0.609 ± 0.003**	Moderate	< 0.001
Moderate (580–3249 cpm)	8.5% (-1.4%)	93.4%	**0.603 ± 0.004**	Moderate	< 0.001
Vigorous (> = 3250 cpm)	0.4% (-0.7%)	99.2%	0.456 ± 0.016	Moderate	< 0.001
MVPA (> = 580 cpm)	8.9% (-2.0%)	94.1%	**0.669 ± 0.004**	Substantial	< 0.001

^1^Related-samples McNemar test (binomial distribution).

^2^CPM conversion equation [sqrt(AG_LFE_) = 0.94 x sqrt(AC) + 17.87] applied in conjunction with default ActiGraph cut-points. Equation calculated from non-zero data only.

^3^Optimal cut-points for predicting ActiGraph intensities with LFE filter activated.

Compared to the AG_NORM_ filter, the AG_LFE_ filter increased the difference between the two devices across the three protocols for the frequency of measured sedentary (absolute difference increase; 3.70%), light (2.93%), moderate (2.50%), vigorous (0.13%), and MVPA (0.87%). The protocol which showed the strongest agreement for MVPA also differed by filter; Protocol 2 showed the highest agreement for the normal filter (95.0%; κ = 0.666 ± 0.004) while Protocol 3 proved the strongest when using the LFE filter (94.1%; κ = 0.669 ± 0.004).

## Discussion

The ability to compare physical activity outcomes measured from different accelerometer-based motion sensors is vital for the assimilation of physical activity information, understanding health outcomes, and developing supportive policy. In this study, we assessed the convergent validity of two popular accelerometers by developing three CPM threshold conditions. Although newer devices featuring wearable technology are regularly being released, many large studies have utilized the AC and AG accelerometers and continue to do so prospectively. Knowing how to adjust these analyses appropriately will facilitate a consistent evidence base.

Our results show that the average CPM recorded by the AG_NORM_ (596 ± 203) and the AG_LFE_ (662 ± 219) were higher than the AC (236 ± 94), reinforcing they are substantially different, and that direct comparisons should be treated with caution. These results are supported by Paul, Kramer (11) who demonstrated the AG (model 7164) detected significantly more daily CPM (216.2 ± 106.2) than the AC accelerometer (188.0 ± 101.1). Similarly, Straker and Campbell (12) found the AG (model GT3X) recorded more CPM in the vertical plane (377.5 ± 977.4) compared to the AC (293.7 ± 977.4). Although both of these previous studies have attempted CPM conversion between devices, they did not investigate how device-specific CPM thresholds may rectify these differences.

When determining adherence to physical activity guidelines, it is important to correctly classify time spent in MVPA. The device-specific thresholds in Protocol 1 (normal filter) resulted in a 5.1% difference in estimated MVPA. The second and third protocols reduced this difference to 1.9% and 2.3%, respectively. These results are important when considering a meaningful difference in MVPA. In a meta-analysis comprising of 99,000 participants, Conn et al [18] reported that physical activity interventions increased MVPA by an average of 14.7 minutes per week (2.1 minutes per day). It is apparent that correctly identifying small differences is important.

The LFE filter present on the ActiGraph enables lower intensity movements to be detected. The results in Tables 2 and 3 demonstrate that the LFE filter exacerbated the discrepancy between devices, particularly for sedentary, light, and moderate intensity physical activity across all three protocols. Considering the AG tends to record higher CPM compared to the AC when using the normal filter, this result is somewhat expected. This has potential implications since it has been recommended that researchers use the LFE filter to improve comparability between the GT3X and earlier AG models [14]. Researchers should be aware that while this choice may improve the comparability between AG models, it may reduce the comparability with other brands.

When broken down into intensity categories, the two with the most visible discrepancy were sedentary and light intensity physical activity. The device-specific thresholds used in Protocol 1 both define sedentary activity as less than or equal to 99 CPM, yet the AC recorded 20% more sedentary time, and 16% less light intensity time compared to the AG (normal filter). This suggests that the AG categorises a larger portion of lower-intensity movements as light intensity, while the AC classes these as sedentary, at least at the 99 CPM threshold. The differentiation between sedentary and light intensity activity is important for studies focusing on patterns of sedentary behaviour, particularly for assessing breaks in sedentary time [19]. The second and third protocols produced marked improvement in sedentary and light classification, and, at face value, may suggest that one of sedentary thresholds used in Protocol 1 needs to be adjusted.

These observations lead to an important question: Do thresholds perform equally in all populations? Threshold development studies normally focus on one age subgroup (e.g., adults, adolescents, pre-schoolers) and develop thresholds specifically for these groups [20–22]. Researchers typically have a number of options when selecting a set of thresholds for a study, and generally choose those which have been validated in a sample which closely resembles their own (in terms of age). The validity of these thresholds are often taken lightly and unquestioned. We suggest that a set of thresholds developed specifically for adults does not necessarily mean they will perform well for *all* adult samples. It may be that thresholds perform better in samples which closely resemble the sample from which they were developed.

While our results demonstrate that the AC tends to record lower CPM compared to the AG, there appear to be distinct clusters of activity where the opposite is true: the AC measured higher CPM compared to the AG at intensities greater than 10000 CPM (see Fig 1). It is possible this was caused by the ‘plateau phenomenon’ present in AG accelerometers, where higher frequency signals are eliminated by the band-pass filter [23]. Nonetheless, this still raises questions about what movements were performed during these periods of activity. Free-living activities include movements other than walking and running at varying degrees of intensity (activities that are commonly used to develop CPM thresholds). It is likely that CPM thresholds developed purely via treadmill protocols fail to take into account activities of this nature. Variability in CPM output is influenced the magnitude (m·s^-2^) and frequency (Hz) of the detected acceleration. Esliger and Tremblay (9) demonstrated the variability in the AG output was related to the frequency of acceleration, while the variability in the AC output was related to the magnitude of acceleration. It is possible that particular free-living activities may have certain frequency-magnitude profiles, dissimilar from typical gait movements, which may increase or decrease the discrepancy between devices. This has implication for treadmill-based cut-point methodology in general, and that is, no matter how rigid development is, cut-points may never be universally applicable due to these reasons.

While the sample in the current study was sufficient to investigate discrepancies between the two accelerometer models, replication in a larger sample from a different population would increase confidence in the generalisability of the results. Another potential limitation is the assumption that devices of the same brand were calibrated similarly, and thus exhibited acceptable inter-monitor reliability. Although both the AC and AG are known to have reasonably high intra-instrument reliability, they still show variations in output when exposed to the same conditions [9]. It is possible that the differences between the AC and AG accelerometers may have been reduced or exacerbated depending on how well the devices of the same brand represented each other. It should also be noted that these results apply to waist-mounted accelerometers only; wrist-worn devices may show significantly different agreement patterns.

## Conclusions

Identifying small differences in physical activity outcomes is important for evaluating the effectiveness of physical activity interventions. Accelerometers have become an integral part of physical activity research, yet data comparability between devices is not without complication. We demonstrated that regression models developed to rectify differences between the Actical and ActiGraph accelerometers improved data agreement. In practice, this means that application of the regression equations in Protocol 2 or the cut-point adjustments in Protocol 3 should result in better alignment of existing AC and AG datasets. However, the outcomes of this study may not be consistent across all populations. Researchers should therefore continue to be aware of the inherent limitations of count-based physical activity assessment when reporting and interpreting study findings.

## Supporting information

S1 Table (XLSX)
